# Trauma, identity and transition: evaluation of the RESLEAPS programme for retiring senior police officers

**DOI:** 10.3389/fpsyt.2025.1722156

**Published:** 2026-02-23

**Authors:** Ceri R. Jones, Stuart Noble, Sylvia Delpratt

**Affiliations:** 1 School of Psychology and Vision Sciences, University of Leicester, Leicester, United Kingdom; 2 Faculty of Business and Law, Birkbeck, University of London, London, United Kingdom; 3 Police Leavers Partnership, Southport, United Kingdom; 4 Faculty of Computer Science, Engineering & Media, Institute for Sustainable Futures, De Montfort University, Leicester, United Kingdom

**Keywords:** police, resettlement programmes, identity continuity, SIMIC occupational trauma, retirement

## Abstract

**Introduction:**

Police officers are repeatedly exposed to trauma throughout their careers, with senior leaders experiencing the cumulative burden of critical incidents, organisational stress, and public accountability. As many retire in midlife, the transition can be psychologically disruptive, impacting identity, mental health, and future employability. This study evaluated a novel RESilient LEAders – Police Superintendents (RESLEAPS) resettlement programme designed to support psychological wellbeing, resilience and future employability during this transition.

**Methods:**

This evaluation combined pre-programme psychological screening and qualitative interviews. Forty-seven participants completed standardised mental health measures, including the GAD-7 (anxiety), PHQ-9 (depression), and TSQ (trauma symptoms). Additionally, 17 participants took part in semi-structured interviews evaluating the programme and exploring emotional, identity, and vocational aspects of retirement. Reflexive thematic analysis was used to identify salient themes.

**Results:**

At baseline, 17.02% of participants met the clinical threshold for moderate anxiety and 23.4% for trauma-related symptoms. Thematic analysis generated six interrelated themes: (1) High Pressure and Workload, including a pervasive sense of jeopardy; (2) Public Perceptions and Politicisation of Policing; (3) Psychological Impacts of 30 Years’ Service, including cumulative trauma and fatigue; (4) Loss of Valued Role, Identity and Sense of Self; (5) Safe Space to Share Vulnerabilities through peer reflection; and (6) Reframing the Retirement Mindset toward renewed purpose and employability.

**Discussion:**

Findings highlight retirement is both a point of vulnerability and an opportunity to strengthen resilience in police officers. Trauma-informed, identity-based resettlement interventions like RESLEAPS can support psychological resilience, confidence, and continued employability beyond policing. Future research should examine long-term wellbeing and vocational outcomes of these programmes.

## 1 Introduction

National data show that police officer attrition has risen steadily in recent years. In 2023/24, 9,366 officers left the service in England and Wales, equating to a 6.2% (1) leaver rate – higher than the average public sector turnover rate of 4.8% (2). Of these leavers, 45% retired and 33% resigned voluntarily (1). In comparison, the UK’s all-industry average turnover is approximately 13–14% (3), suggesting that while police retention remains comparatively strong, the rise in voluntary resignations reflects growing strain within the service.

The transition from a long policing career into retirement is a significant life event that can have profound psychological implications. Leaving policing can lead to increased stress, loss of identity, and heightened risks of mental health issues, particularly for officers with extensive trauma exposure (4, 5). Policing involves cumulative exposure to distressing events rather than a single incident, many officers experience symptoms consistent with Complex Post Traumatic Stress Disorder (cPTSD) as recognised in the ICD-11 (6). A large UK study found that one in five officers met the diagnostic criteria for post-traumatic stress disorder (PTSD) or Complex PTSD (c-PTSD) (7). Estimates of probable PTSD among UK police officers range from 20–30% (8, 9), considerably higher than the 4–5% prevalence in the general population (10). The highly structured, hierarchical nature of policing fosters a strong professional identity, making the transition particularly challenging (11). Many officers also struggle with the loss of camaraderie and peer support networks post-retirement (12).

### 1.1 Police superintendents

Police Superintendents represent the senior operational leadership tier within UK policing, typically one rank below Chief officer level (Chief Constable, Deputy Chief Constable, Assistant Chief Constable, etc.). They hold substantial managerial, strategic, and public accountability responsibilities, often commanding entire policing areas or specialist departments. Depending on their posting, Superintendents may serve as Divisional Commanders overseeing local policing across multiple stations and communities, or as Heads of Specialist Functions such as Crime and Intelligence, Professional Standards, Public Protection, Roads Policing, Counter-Terrorism/Special Branch, or Community Safety.

The scale of these roles is considerable: a Superintendent may have direct line management of 200–500 officers and staff, control budgets exceeding £10–20 million, and be responsible for major operational decisions, critical incidents, and inter-agency partnerships. Their duties involve balancing strategic planning, performance management, and public accountability with high personal exposure to risk, trauma, and scrutiny.

These complex and competing pressures leave them vulnerable to mental health issues stress and burnout as demonstrated by the Superintendents’ Resilience Survey (13), over three quarters (77%) of superintendents reported an excessive workload. In addition, 73% of respondents reported that their workload had increased in the previous 12 months with high levels (63%) of reported mental health issues, stress, low mood and anxiety.

For many the rank of Superintendent represents the highest level of career attainment, and such senior ranks comprise a larger proportion of older officers approaching retirement (14). Senior officers, with approximately 30 years’ service, will have been exposed to an estimated 900 traumatic events in their careers (15). Whilst promotion to Superintendent may reduce frequency and direct exposure to traumatogenic events and material, the cumulative legacy of repeated exposure can heighten the risk of developing anxiety, depression and Post Traumatic Stress (PTSD) symptoms exacerbated by occupational related psychological hazards such as high increased work stressors and burnout (16). Organisational factors such as high workload and a macho organisational culture can increase the likelihood of poor mental health in this occupational group (17). With lengthy, collegiate service, bonds between senior officers are strong, and loss of membership from this group can be challenging (18). Retirement can lead to strong emotions such as anger, disappointment, financial issues, family conflicts and the loss of professional networks (19).

### 1.2 Policy and practice context

Historically, police officers served approximately 30 years before retiring with a full pension. Meaning for the majority who joined in their late teens early twenties they will be retiring in their early fifties. More recent legislative changes such as the introduction of the 2015 Police Pension Scheme, have changed this trajectory. The new scheme raised the normal pension age to 60, allowing flexible retirement options from the age of 55, albeit with actuarially reduced pensions (20).

Our research on police leavers found that the majority planned to continue working, with 61.4% intending to fully retire between 65 and 69 years of age. Yet despite this desire for a second career, a significant majority (76.5%) reported that their employing police force provided no resettlement support. This lack of structured provision led almost half (48.8%) to seek external assistance independently (21).

Typically, retirement support for police officers focuses on the financial and pension planning aspects there is little focus on the emotional and psychological aspects on leaving the service.

The UK government has recognised the importance of post-service support through the Police Covenant introduced in 2022 aims to ensure that individuals who have served in the police are not disadvantaged as a result of their service. The Covenant commits the government and policing bodies legally to support the physical health, mental wellbeing, and financial security of officers, staff, and their families throughout service and into retirement (22).

Recent UK policy has highlighted the risks of rising economic inactivity among adults over 50, particularly following COVID-19 (23). Without structured support, many individuals disengage from the labour market, with consequences for mental health, social connectedness, and reliance on public services (24).

Mental ill-health compounds this risk. The Centre for Mental Health (25) estimates the national cost of poor mental health at £119 billion annually. With around one in five officers meeting the diagnostic criteria for PTSD or complex PTSD (7). Investing in trauma-informed resettlement can therefore yield wider public value by supporting wellbeing, enabling continued employment, and reducing pressure on nationally publicly funded health services.

### 1.3 The Social Identity Model of Change

The Social Identity Model of Identity Change (SIMIC) (26) provides a theoretical framework to understand the psychological challenges of career transitions, including police retirement. SIMIC suggests that individuals who transition from one role to another face significant identity disruptions, particularly if they lack multiple, flexible social identities. The theory posits that identity continuity, and the development of new group memberships are necessary for maintaining psychological wellbeing during life transitions. Previous research applying the SIMIC has shown that structured support interventions help individuals retain their sense of identity post-retirement (27). In policing, where professional identity is deeply embedded in hierarchical organisational structures and team identity, retirement can be particularly destabilising. The SIMIC model suggests without a structured transition, officers may struggle to reconstruct their identity, leading to increased stress, anxiety, and social isolation (28).

In the UK, policing is often a 30-year career with superintendents joining at 18 then retiring in their early 50s. Many wish to continue to work or ‘rejoin’ policing in civilian roles or seek other roles in other industries. An individual’s self-identity can be reinforced by their social identity within an organisation. Police culture creates a strong sense of belonging identify amongst its members (29) hence it can be challenging for those leaving to find employment in other industries organisations and roles, that reflect their values and create a renewed sense of purpose and comradery with colleagues. This finding has also been reflected in other industries that fosters strong organisational identity and sense of belonging such as the armed forces (30). Where transition out can be a source of anxiety status loss and lead to lack of purpose and loneliness (31). The SIMIC model applied in the armed forces highlighting the need for transition support for groups with strong occupational identities transitioning into civilian life to protect their wellbeing (32) identity adjustment and employability prospects (33).

### 1.4 The RESLEAPS programme

The RESilient LEAders – Police Superintendent (RESLEAPS) resettlement programme was developed to provide comprehensive support for police superintendents as they transition from service into retirement. Designed and delivered collaboratively by a chartered psychologist and a retired chief superintendent, the programme aimed to mitigate psychological distress, foster resilience, and support individuals in preparing for a professional transition into a new career. The material drew on the SIMIC framework, reflective peer support and adopted a trauma-informed approach, recognising the impact of trauma and the need for safety, trust, and empowerment within the sessions (34, 35). The programme was designed to complement existing police pre-retirement provision, which has traditionally focused on pensions and financial planning. In contrast, it offers a trauma-informed, psychologically grounded approach that promotes wellbeing, identity transition, and career re-engagement.

#### 1.4.1 Programme objectives

- To provide a structured framework for officers to plan their transition effectively.
- To support officers to proactively manage the mental health and emotional wellbeing.
- To support officers in identifying and articulating their transferable skills.
- To create a safe and supportive space to share concerns with peers.

#### 1.4.2 Programme structure and content

RESLEAPS consists of three core components, each tailored to key aspects of transition:

1. Practical Preparation: A transition timeline to help officers plan key milestones, ensuring officers have ample time to prepare.
2. Personal Identity and Resilience: Addressing the emotional and psychological challenges of leaving policing, incorporating psychoeducation, resilience-building strategies, identifying values and purpose exercises.
3. Professional Career Planning: Guidance on CV development, job search strategies, interview skills, and leveraging social networks for career opportunities.

#### 1.4.3 Programme delivery

RESLEAPS was delivered across five UK police forces via in-person facilitation. The delivery team included a chartered psychologist, retired superintendent and assisted by a research associate Forces included large metropolitan forces and medium size regional forces. Employees ranged from several thousand to tens of thousands of officers, reflecting a broad spectrum of leadership experiences and organisational structures. The programme ran between August 2022 and September 2023. Superintendents and Chief Superintendents who were 2 years pre-retirement were invited to take part. A total of 69 (53%) Superintendents and Chief Superintendents attended at least 1 session. Each force received the same content, but the delivery format ranged from; six, two-hour modular sessions, four half day sessions or 2 full day sessions. The delivery was tailored to the needs of participating forces and officer availability. The programme was designed to encourage peer support, creating a safe space for participants to reflect, share concerns, experiences, and strategies for a successful transition.

## 2 Methods

### 2.1 Study design

A pre-programme quantitative assessment was undertaken to understand the clinical profiles of programme participants. A qualitative post programme evaluation was undertaken to explore the experiences of participants who took part. The evaluation focused on understanding the challenges, benefits, and overall impact of the programme on retiring police superintendents. In addition, an indicative cost–benefit analysis (CBA) was conducted to explore the potential economic value of the programme using publicly available data on re-employment, taxation, and mental health service costs. As long-term employment outcomes were not captured, the analysis draws on comparable data from military resettlement programmes.

### 2.2 Participants and recruitment

For the qualitative evaluation, all programme participants (N = 69) were invited to take part in interviews. Invitations were sent via email between June 2023 and July 2024, and 17 participants who had engaged with the RESLEAPS programme agreed to participate. Participants were purposefully sampled (Patton, 2002) to ensure in-depth perspectives on the programme’s impact.

All programme participants (N=67) were invited to take part in an online pre-screening questionnaire, 68.1% (n=47) completed standardised psychological measures assessing depression (PHQ-9), anxiety (GAD-7) and trauma symptoms (TSQ).

### 2.3 Data collection and analysis

#### 2.3.1 Quantitative

The psychological measures were scored according to standard criteria. PHQ-9 and GAD-7 scores ≥10 indicated clinically significant symptoms. TSQ: A cut-off of ≥6 indicated significant posttraumatic stress symptoms. Descriptive statistics were used to summarise mean scores and clinical cut-offs. Independent t-tests were conducted to examine gender differences in anxiety and trauma symptoms.

In addition to the psychological and qualitative evaluation, an indicative cost–benefit analysis was conducted to explore the potential economic value of the RESLEAPS programme. This estimate draws on publicly available figures for employment income, taxation, and mental health service costs to illustrate possible returns on programme investment.

#### 2.3.2 Qualitative

Data was collected through seventeen in-depth interviews, conducted via Microsoft Teams between July 2023 and October 2024. A semi-structured interview guide was used to explore participants’ experiences, focusing on challenges of the transition, psychological impacts and overall programme effectiveness. Interviews ranged from 43 minutes to 112 minutes and were recorded with participant consent.

Interviews were transcribed verbatim and analysed using Braun and Clarke’s (36) six-step framework. The analysis was conducted using manual coding utilising an inductive approach. Initial coding was conducted by the lead author followed by a review and refinement of themes to ensure consistency. Intercoder agreement was established through discussions among the research team to validate key themes.

#### 2.3.3 Indicative cost–benefit analysis

In addition to the psychological and qualitative evaluation, an indicative cost–benefit analysis was conducted to explore the potential economic value of the RESLEAPS programme. This estimate draws on publicly available figures for employment income, taxation, and mental health service costs to illustrate possible returns on programme investment.

### 2.4 Ethical considerations

Ethical approval was obtained from the University of Leicester Ethics Committee. Informed consent was secured from all participants before data collection. Participants were assured of confidentiality, anonymity, and their right to withdraw. Pseudonyms were assigned to maintain anonymity, and all data were stored securely in compliance with GDPR regulations data policies.

## 3 Results

### 3.1 Psychological profile of participants

A total of 47 pre-programme participants (Male: 28, Female: 19) completed psychological measures assessing depression, anxiety, and trauma-related symptoms. The mean age of participants was 51.96 years (Male: 51.71 years, Female: 52.32 years), and the mean length of service was 29.11 years (Male: 29.04 years, Female: 29.21 years).

As shown in **Table 1**, none of the participants met the clinical cut-off (≥10) for moderate depression, indicating a low prevalence of depressive symptoms in this cohort. However, 17.02% of participants met the clinical threshold (≥10) for moderate anxiety, suggesting that a notable subset experienced heightened anxiety prior to programme participation. Additionally, 23.4% of participants met the clinical cut-off (≥6) for trauma-related symptoms, indicating that nearly a quarter of the cohort reported significant trauma related distress. These findings indicate higher levels of psychological distress than population norms. Around 19% of UK adults experience moderate-to-severe anxiety or depression (37), compared with 65% of serving officers (38). Likewise, 4–5% of adults meet criteria for PTSD (10), versus 20–30% of UK police officers (7, 8). This pattern reflects a markedly greater mental health burden within policing relative to the general public.

**Table 1:** Psychological profile of participants.

Measure	% Above clinical cut-off	Mean score (total sample)	Mean score (male)	Mean score (memale)	t-statistic	p-value
PHQ-9 (Depression)	0	0	0	0		
GAD-7 (Anxiety)	17.02	5.77	6.71	4.37	1.78	0.08
TSQ (PTSD Symptoms)	23.4	3.3	2.89	3.89	−1.16	0.25

### 3.2 Gender differences in psychological distress

The cohort comprised 28 male and 19 female officers, reflecting national data showing the gender imbalance typical of UK policing, particularly at senior ranks, where female representation declines with seniority (1). The sample’s composition is therefore consistent with wider workforce patterns. A comparison of clinical scores by gender revealed some differences in anxiety and trauma symptoms. Male participants reported higher anxiety levels (M = 6.71) compared to female participants (M = 4.37), though the difference was not statistically significant (t(45) = 1.75, p = 0.09). Conversely, female participants had higher trauma-related symptom scores (M = 3.89) compared to male participants (M = 2.89), but this difference was also not statistically significant (t(45) = −1.19, p = 0.24).

### 3.3 Qualitative results

Six themes were identified; challenges of retirement included i) High Pressure & Workload, ii) Public Perceptions & Politicisation, iii) Psychological Impacts of 30 Years’ Service, and iv) Loss of Valued Role, Identity & Sense of Self, while programme benefits included v) Safe Space to Share Vulnerabilities and vi) Reframing Retirement Mindset.

#### 3.3.1 High pressure & workload

Programme participants discussed the high pressure and workload of their roles. With a lack of formal retirement or resettlement programmes, often individuals were working up until their retirement date and described it as falling off a *‘cliff edge’*.

*“Years ago in policing when I had first started, when you got to the to the rank of senior officer, it was very much a wind down process. But I think over time now the role of senior officer quite rightly is become more and more important and very visible role and it’s you almost a ramp up towards the end of your service and you work longer hours and you’re responsible for far more risk than ever and and it’s you know to suddenly kind of stop.”* (Suzie)

##### 3.3.1.1 Sense of jeopardy

Being on call up until retirement increased their sense of jeopardy. Participants expressed concerns they may be involved in a serious incident that could impact or delay their retirement. Participants discussed the benefits of coming off call 6 months before retirement, but there was sense of guilt of pushing the burden onto colleagues. For some this was not possible due a lack of superintendents to cover on call rotas.

*“I remember a couple of times people got involved in serious incidents, you know, with a week to go and they thought the pensions are gonna be in jeopardy”* (Tom)

##### 3.3.1.2 High levels of organisational commitment

Participants described high levels of engagement and commitment to policing, having made sacrifices throughout their career. Prioritising the job first before family and the role becoming all consuming. Individuals felt they wanted to complete the current projects they were working on and leave them in a good place for others to pick up and continue, going *‘the extra mile’* till the end to leave a positive legacy.

*“I’ve been a very engaged employee. I think I’ve had four days sick and 30 years. I’ve always gone the extra mile. I was on call 24/7 most of my career almost every day because of the nature of work”* (Sammuel)

#### 3.3.2 Public perceptions & politicisation

The current climate of policing was making participants consider retiring earlier than originally planned. Participants described the *‘politicisation’* of policing as moving away from the traditional values. Being directly accountable to politicians who are *“only interested in getting re-elected and getting votes*” as challenging.

*“I think that I think the big problem with policing was when they politicised it, so that’s that has in my mind destroyed police and in all honesty so … policing should be without fear or favour doing what’s right for the public and to be trusted to deliver”* (Sammuel)

##### 3.3.2.1 Loss of policing values

Participants discussed a move away from core policing values of *‘protecting the public and fighting crime’*. Core values such a policing by consent were being eroded. The job had changed since they joined 30 years ago, and this was influencing their decision to leave.

*“The police are the public and we police by consent and I think that’s been eroded. So, some difficulties there.”* (Arthur)

#### 3.3.3 Psychological impacts of 30 years’ service

Individuals described how they had seen ‘*horrendous things’* throughout their career and the impact of cumulative traumatic exposure. They were concerned how these might impact them when they retire. At a senior level there were also the challenges of the organisational and operational stressors. There was an acknowledgement of the psychological impacts of their long tenure and a need to decompress.

***“****What is the impact of 30 years of stress related incidents on you and your emotions and your mental wellbeing, but also how are you going to engage with the emotional side of things?”* (Elliot)

#### 3.3.4 Loss of valued role, identity & sense of self

Participants disclosed their anxiety approaching retirement in relation to their loss of role and identify. They described themselves as being *‘institutionalised’* and that being a police officer is not only what they do but who they are. Retiring from role would leave a massive *‘void’*. Participants described the symbolism of handing over their warrant card that they have had for 30 years. Others were worried about loss of colleagues and peer networks post-retirement.

*“I think it is quite deeply ingrained, actually, and 30 years of of doing it. And I think that inevitably becomes part of who you are, what your response to things is and the pros and cons of that, which you know there’s a lot of pros, but there are some cons of that as well. So yeah, so it’s gonna be an interesting transition”* (Mary)

##### 3.3.4.1 Low confidence & competence

Participants described their lack of confidence in applying for roles outside of policing. They were worried they might not have the right skills and qualifications. Participants will have gone through several promotion processes to get to the seniority of rank. Yet most hadn’t written a formal CV or been for an external interview since joining the service at 18 and acknowledged that these processes were markedly different from the internal process they were familiar with.

*“Yeah, sometimes it’s not your competence. It’s who you’re up against, you know, so … That’s not to be. I’ll move on to that. But it did, it did give me a stumble in my confidence and thinking. Ohh Christ, what I’m, you know, am I gonna be driving a van round Tesco delivery?”* (Joe)

#### 3.3.5 Safe space to share vulnerabilities

Participants described how the RESLEAPS programme gave them a safe space to share their emotional vulnerability towards retirement with peers. There was a recognition of shared experiences and vulnerabilities. This safe space allowed participants to work through some of their challenges together and learn from each other, fostering stronger social support.

*“I think hearing just from others really. So that conversation in the room that was generated, and you know the inputs that were given. Around what people were planning to do or not planning to do, or I suppose understanding that others are as confused as me was useful because then we could work through options together. that feeling of not being alone”* (Philip)

##### 3.3.5.1 Peer support

The programme included peers and veterans, with time and space for reflection. This was seen as particularly valuable. Some described how it was *‘lonely being a leader’* with a lack of peer network at senior levels. There was recognition that others were experiencing the same emotions and challenges creating a shared understanding and trust.

*“Best thing was just understanding other people. Having the same thoughts as you, and although they might not come to the same conclusion, they were considering all the same things as you. I had the same fears as you.”* (Jimmy)

#### 3.3.6 Reframing the retirement mindset

Programme participants shared how the programme enabled them to be more psychologically prepared for retirement, develop cognitive flexibility and reframe retirement as a new opportunity. At superintendent rank there is a lack of programmes to support those retiring. This programme helped them think about the practical elements such as, CV writing and transferable skills but also to emotionally and cognitively prepare for that transition. The programme helped them reframe leaving as a positive opportunity and be more optimistic rather than feeling fear and anxiety,

*“There’s a lot of … financial advisors rather and you know, wealth management companies. That’s great but your programme is different because it was, it was around some practical stuff, which is good for CV’s etc, but mostly around I think the, um, the mental side for me and support which came from both having the group.”* (Arron)

*“I feel as strong mentally as I can be to move forward and your programme really help. So yeah, I don’t say that lightly”* (Levi)

##### 3.3.6.1 Conscious transition

Participants described how the programme helped them on their retirement journey. It allowed them to proactively manage that transition and take practical steps towards their next chapter.

*“But I’m consciously scoping and looking at opportunities rather than being on the back foot and just head in the sand. I’m working. I think I’ve now lifted my game”* (Albert)

*“I certainly felt less stressed coming away from the sessions I thought. Ohh right. OK, you know, clarity.”* (Fay)

##### 3.3.6.2 Transferable skills translation

Participants feedback that the programme helped them understand and translate their transferable skills into new opportunities that met with their values. They felt more confident in being able to market themselves to other industries.

*“So, to actually have inputs on writing a CV or understanding how your skills translate into those CPD qualifications and things was really fascinating. You know, really useful”* (Bill)

### 3.4 Indicative cost–benefit analysis

In addition to assessing psychological wellbeing and qualitative experiences, an indicative cost–benefit analysis was conducted to explore the potential economic value of the RESLEAPS programme. Although no direct employment data were collected, assumptions were made based on comparable evidence from military resettlement programmes, given the structural and psychosocial similarities between military veterans and senior police officers. **Table 2** outlines estimated economic returns based on mid-range assumptions for post-retirement employment and avoided healthcare costs.

**Table 2:** Indicative cost–benefit analysis.

Category	Estimated annual public benefit	Notes
Programme Cost	£240,000 (one-off)	Funded by Movember; pilot delivery and evaluation
Reduced NHS service usage	~£35,000–£70,000	Based on 23% trauma and 17% anxiety prevalence (25)
Increased PAYE contributions	~£735,000	Assuming 35 of participants are re-employed at £70k average salary
Reduced pension drawdown pressure	Not directly monetised	Longer economic activity may delay full pension reliance
Retained leadership expertise	Not directly monetised	Harder to quantify, but relevant to public sector value
Total Estimated Benefit	~£770,000–£805,000	Based on partial monetised benefits only

*Benefits are annualised; programme cost is a one-off.* Estimates are based on publicly available data including MOD’s Career Transition Partnership outcomes, Forces in Mind Trust evaluations, and NHS mental health service cost estimates from the Centre for Mental Health (25, 39–41). 35 of the 69 RESLEAPS participants (50.7%) were assumed to gain employment, reflecting military comparators.

## 4 Discussion

The findings from the pre-screening and post-evaluation of the RESLEAPS resettlement programme provide valuable insights into the experiences of senior police officers transitioning out of service. Thematic analysis identified key challenges associated with the retirement transition, including High Pressure and Workload, Public Perceptions & Politicisation, Psychological Impacts of 30 Years’ Service, Loss of Valued Role, Identity & Sense of Self. Programme benefits included a Safe Space to Share Vulnerabilities and Reframing the Retirement Mindset. These themes align with existing literature on career transitions in policing, highlighting the psychological impacts of long-term service with high levels of trauma exposure and strong role identity (e.g., 11, 19).

### 4.1 Psychological distress in retirement transition

The quantitative analysis of the pre-screening questionnaire provides evidence of the psychological distress in retiring superintendents, particularly in relation to anxiety and trauma symptoms. While depression levels were low, with no participants meeting the clinical threshold for moderate depression (PHQ-9 ≥10), 17.02% of participants reported clinically significant anxiety symptoms (GAD-7 ≥10), and 23.40% met the clinical cut-off for posttraumatic stress symptoms (TSQ ≥6). These findings suggest that, although retiring senior officers may not exhibit high levels of depressive symptoms, anxiety and trauma-related distress remains a significant issue during the transition period.

These results reinforce previous research demonstrating that police officers experience elevated risks of PTSD and anxiety due to cumulative service-related trauma exposure (9, 42). Drawing on research from the armed forces associated identity loss, and uncertainty about the future are likely to exacerbate these mental health risks (33, 43).

### 4.2 Gender differences in psychological distress

Although gender differences in anxiety and trauma symptoms were observed, these differences were not statistically significant. Male participants reported higher anxiety levels (M = 6.71) compared to female participants (M = 4.37), though this difference approached significance (t(45)=1.75, p=0.09). Conversely, female participants had higher trauma-related symptom scores (M = 3.89) compared to male participants (M = 2.89), though this difference was also non-significant (t(45) = −1.19, p=0.24). While the small sample size may have limited statistical power, these findings highlight potential gender-based variations in psychological distress, warranting further exploration in future research.

### 4.3 Demands of the role

Participants described increasing workloads and pressures leading up to retirement, often resulting in a sudden and abrupt transition. The metaphor of “falling off a cliff edge” was used to illustrate the stark contrast between high-intensity roles and the cessation of duties post-retirement. The lack of structured transition support was a significant concern, aligning with research on the need for phased retirement strategies in policing (12, 44). Additionally, anxiety about potential involvement in critical incidents before retirement led some participants to advocate for a six-month phased withdrawal from frontline duties.

Public perceptions and the increasing politicisation of policing were also identified as major stressors, contributing to earlier-than-anticipated retirement decisions. Participants noted a shift in core policing values, creating a disconnect between their professional identities and police service expectations. These findings are consistent with prior findings on moral injury and value misalignment in policing (29, 45).

### 4.4 Identity transition

For senior police leaders, policing was not merely a job but a core component of their identity, resulting in high levels of organisational commitment, making the transition and loss of role particularly challenging for their wellbeing. Retirement from policing is a point of vulnerability with rates of suicide 3 times higher than that of the general population (46). Retirement was described as not only the end of a career but also the loss of a core identity. The symbolism of surrendering a warrant card was significant for many participants, underscoring the depth of institutional identity within policing. This aligns with previous research findings that the police service creates a strong sense of in-group identity between officers (47). However, the RESLEAPS programme played an important role in facilitating self-reflection and skill translation, helping officers reframe their professional experience and consider future opportunities outside of policing. Findings parallel with research on career transitions in policing and military professions, where structured support is important for mitigating psychological distress (31). The loss of professional identity, particularly in roles with high-status affiliations, has been linked to increased depression and anxiety in retirement (26). Similar to military veterans (48), retiring senior police officers could benefit from structured programmes that address identity reformation and peer support networks.

Furthermore, the transition brought concerns about loss of confidence and competence in securing post-retirement employment, with many officers unfamiliar with modern job application processes. Police superintendents found it difficult to identify and translate their police skills and experience to civilian roles in other industries. Similarly, research has found that for those leaving the armed forces, military skills often do not directly translate to civilian job roles and face difficulties in articulating their skills in a non-military context (49). Superintendents fed back that the RESLEAPS programme helped them confidently translate their skills and experience using language that was more easily understood by those in the civilian job market.

### 4.5 Trauma informed support

Findings suggest that structured resettlement programmes should integrate trauma-informed psychoeducation to help officers manage cumulative service-related stress and mental health issues, as 17.02% of participants exhibited clinical anxiety symptoms and 23.4% met the threshold for posttraumatic stress symptoms. The programme followed trauma informed guidelines (50) and includes exercises aimed at improving psychological wellbeing and optimism about the future, such as the “Best Possible Self” (BPS) intervention. This involves envisioning one’s best possible future self after everything has gone as well as possible and writing about it (51). The BPS is an effective intervention to improve optimism, wellbeing, mood and depression (52). Based on the trauma recovery literature and best practice guidance (34) the programme also includes psychoeducation on a range of factors encompassing cognitive, behavioural, and existential constructs that have been identified as supporting resilience in response to trauma exposure (53).

Building on the Social Identity Model of Identity Change (SIMIC), we propose that police retirement be conceptualised under an extended framework: SIMC-T (T=Trauma). This addition acknowledges that identity transition for police veterans is uniquely shaped by traumatic exposure and the psychological toll of long-term service, distinguishing it from other career transitions. This aligns with previous research demonstrating that police officers and military veterans experience elevated risks of PTSD and other mental health challenges due to cumulative service-related trauma (9). Resettlement programmes should integrate trauma informed approaches that can support identity reconstruction and mitigate the psychological impacts of long-term service (19).

### 4.6 Peer networks and social support

Furthermore, the study reinforces the importance of peer support networks in retirement transition. The RESLEAPS programme facilitated shared identity maintenance through peer discussions, helping participants reframe their experiences and navigate post-retirement challenges together. This aligns with research on social reconnection and resilience-building in high-stress professions (12, 27). Participants also found structured networking and career transition guidance particularly valuable, supporting findings that officers often struggle to articulate their transferable skills in non-policing contexts (49).

### 4.7 Indicative cost–benefit analysis and public value

While the core aim of this study was to explore the psychological and identity impacts of retirement, indicative modelling suggests that resettlement support like RESLEAPS may also deliver public value. Delivered with £240,000 in charitable funding, the programme supported 69 senior officers across five UK forces. Based on military resettlement comparisons, the estimate that 35 participants (50.7%) gained new employment is conservative.

If each earns £70,000, this equates to £2.45 million in annual earnings and approximately £735,000 in tax revenue, over three times the programme’s cost. Sustained over two years, this would exceed £1.4 million. Additionally, over 40% of participants met clinical thresholds for anxiety or trauma. If resettlement support helped just 28 individuals avoid NHS intervention, it could save between £35,000 and £70,000 in healthcare costs annually.

While further longitudinal research is needed, these figures suggest that structured, trauma-informed resettlement programmes can support both psychological recovery and economic reintegration, yielding substantial public value.

### 4.8 Implications

While the Social Identity Model of Identity Change (SIMIC) has primarily been applied to explain psychological adaptation during transitions such as retirement and redundancy (54), its relevance to employment outcomes remains underexplored. This study suggests that SIMIC-informed interventions, by promoting identity continuity, protecting wellbeing and resilience and may indirectly support employability, particularly in professions where occupational identity is deeply embedded, such as policing.

The RESLEAPS programme demonstrates the value of a structured, trauma-informed approach to this complex transition. Programmes that combine psychoeducational elements, peer reflection, and career planning can support officers in reframing their identities, translating their skills, and preparing for re-entry into the workforce (31). This reflects broader calls for more flexible and dynamic models of retirement that accommodate extended, non-linear working lives (55).

The Covenant emphasises police forces have a duty to reduce disadvantage for those leaving policing (56). It could be argued that the RESLEAPS programme operationalises the intent of the Covenant by focusing on the emotional, identity, and practical dimensions of leaving policing areas often overlooked in traditional pre-retirement provision. From a policing perspective, structured transition support is particularly important given the high prevalence of clinically significant trauma and anxiety in this cohort. Trauma-informed interventions can prevent disengagement from future employment, reduce pressure on occupational health and welfare services, and sustain the contribution of experienced leaders to policing and the wider public sector. Peer support structures and phased retirement pathways may mitigate the psychological disruption of abrupt exit (48) and help maintain role continuity, belonging, and wellbeing (27, 28).

### 4.9 Limitations

This study has a number of limitations. First, it was primarily descriptive, offering a snapshot of the mental health profile of superintendents at programme entry. The absence of follow-up data limits our ability to assess long-term outcomes. Although validated screening tools were used to measure baseline wellbeing, no repeated measures were taken. Future evaluations should adopt longitudinal designs to track psychological and vocational outcomes over time.

Employment outcomes were also not systematically tracked. The cost–benefit analysis presented here draws on assumptions informed by military resettlement research (40, 48), but direct evidence of re-employment rates among participants would strengthen the economic case and represent a direction for future research. Finally, the sample was restricted to senior officers from five forces due to funding restrictions, limiting generalisability.

### 4.10 Future research

Future studies should adopt longitudinal and controlled designs to evaluate the sustained impact of programmes like RESLEAPS on mental health outcomes over time. Comparative research across police ranks and between officers and staff would also be valuable, as different roles may be associated with different psychological risks and needs.

Further investigation into the role of social identity processes during policing transitions is warranted. A SIMIC plus Trauma Informed (SIMIC-T) framework, which integrates identity continuity, identity gain, and explicit attention to trauma, may be particularly relevant in high-risk occupations (27, 28). Cross-sectional studies could explore relationships between trauma symptoms and identity continuity to better understand the psychological mechanisms underpinning successful transitions.

## 5 Conclusion

This study highlights the need for structured, trauma-informed resettlement support for senior police officers navigating retirement transitions. The RESLEAPS programme demonstrates how such interventions can address the psychological, identity-related, and vocational challenges associated with role exit after decades of service. By fostering identity continuity, enhancing psychological wellbeing, and supporting future employability, resettlement programmes can ease the transition into new roles and reduce the risk of mental health-related disengagement. Beyond individual benefits, these interventions may also deliver broader public value through sustained workforce participation, reduced demand on health services, and a return on prior public investment in leadership. As retirement increasingly represents a career pivot rather than a full exit from working life, programmes like RESLEAPS offer a promising model for promoting resilience, reintegration, and long-term economic contribution in high-risk public service professions.

## Data Availability

The raw data supporting the conclusions of this article will be made available by the authors, without undue reservation.
